# SARS-CoV-2 genome variations and evolution patterns in Egypt: a multi-center study

**DOI:** 10.1038/s41598-022-18644-4

**Published:** 2022-08-25

**Authors:** Deena Jalal, Mariam G. Elzayat, Hend E. El-Shqanqery, Aya A. Diab, Abdelrahman Yahia, Omar Samir, Usama Bakry, Khaled Amer, Mostafa ElNaqeeb, Wael Hassan, Hala S. Talat, Hala M. Farawela, Mona S. Hamdy, May S. Soliman, Maha H. El Sissy, Moushira H. Ezzelarab, Sara M. El khateeb, Lamyaa H. Soliman, Sara E. Haddad, Ashraf Hatem, Mohamed S. Ismail, Maha Hossam, Tarek Mansour, Lobna Shalaby, Sonia Soliman, Reem Hassan, Mahmoud Hammad, Ibrahim Abdo, Sameh Magdeldin, Alaa ElHaddad, Sherif Abouelnaga, Ahmed A. Sayed

**Affiliations:** 1 Genomics Program, Department of Basic Research, Children’s Cancer Hospital Egypt 57357, Cairo, Egypt; 2 Egypt Center for Research and Regenerative Medicine (ECRRM), Cairo, Egypt; 3 Department of Pediatric Medicine, Kasr Al-Aini School of Medicine, Cairo University, Cairo, Egypt; 4 Department of Clinical and Chemical Pathology, Kasr Al-Aini School of Medicine, Cairo University, Cairo, Egypt; 5 Department of Pulmonary Medicine, Kasr Al-Aini School of Medicine, Cairo University, Cairo, Egypt; 6 Department of Internal Medicine, Kasr Al-Aini School of Medicine, Cairo University, Cairo, Egypt; 7 Virology and Immunology Department, National Cancer Institute, Cairo University, Cairo, Egypt; 8 Department of Clinical Pathology, Children’s Cancer Hospital Egypt 57357, Cairo, Egypt; 9 Infectious Disease Unit, Children’s Cancer Hospital Egypt 57357, Cairo, Egypt; 10 Department of Pediatric Oncology, National Cancer Institute, Cairo University, Cairo, Egypt; 11 Department of Clinical Pathology, National Cancer Institute, Cairo University, Cairo, Egypt; 12 Molecular Microbiology Unit, Children’s Cancer Hospital Egypt 57357, Cairo, Egypt; 13 Department of Pediatric Oncology, Children’s Cancer Hospital Egypt 57357, Cairo, Egypt; 14 Department of Clinical Pharmacy, Children’s Cancer Hospital Egypt 57357, Cairo, Egypt; 15 Proteomics and Metabolomics Unit, Department of Basic Research, Children’s Cancer Hospital Egypt 57357, Cairo, Egypt; 16 Department of Physiology, Faculty of Veterinary Medicine, Suez Canal University, Ismailia, Egypt; 17 Department of Biochemistry, Faculty of Science, Ain Shams University, Cairo, Egypt

**Keywords:** RNA sequencing, SARS-CoV-2, Viral evolution

## Abstract

A serious global public health emergency emerged late November 2019 in Wuhan City, China, by a new highly pathogenic virus, SARS-CoV-2. The virus evolution spread has been tracked by three developing databases: GISAID, Nextstrain and PANGO to understand its circulating variants. In this study, 110 diagnosed positive COVID-19 patient’s samples, were collected from Kasr Al-Aini Hospital and the Children Cancer Hospital Egypt 57357 between May 2020 and January 2021, with clinical severity ranging from mild to severe. The viral genomes were sequenced by next generation sequencing, and phylogenetic analysis was performed to understand viral transmission dynamics. According to Nextstrain clades, most of our sequenced samples belonged to clades 20A and 20D, which in addition to clade 20B were present from the beginning of sample collection in May 2020. Clades 19A and 19B, on the other hand, appeared in the mid and late 2020 respectively, followed by the disappearance of clade 20B at the end of 2020. We identified a relatively high prevalence of the D614G spike protein variant and novel patterns of mutations associated together and with different clades. We also identified four mutations, spike H49Y, ORF3a H78Y, ORF8 E64stop and nucleocapsid E378V, associated with higher disease severity. Altogether, our study contributes genetic, phylogenetic, and clinical correlation data about the spread of the SARS-CoV-2 pandemic in Egypt.

## Introduction

The severe acute respiratory syndrome coronavirus-2 (SARS-CoV-2) emerged in Wuhan China in December 2019, and since then it has spread to 228 countries and territories constituting a global pandemic causing more than 6 million deaths worldwide^1,2^. Coronaviruses (CoVs) are divided into four subgroups, two of which circulate in mammals; alphacoronavirus and betacoronavirus^3–5^. Over the past 20 years more than seven coronaviruses have crossed the species barrier causing respiratory illnesses with varying severity in humans. The pandemic of COVID-19 poses a lethal challenge due to its aggressiveness, high rate of infectivity, severity of symptoms, poor prognosis, and lack of resources. COVID-19 affects different parts of the body causing a variety of symptoms such as viral pneumonia through infecting the lower respiratory systems, and/or diarrhea and vomiting via the gastrointestinal tract, with varying severity^6^.

Coronaviruses are single-stranded, positive-sense RNA (+ ssRNA) contained in an envelope. The coronavirus genome is approximately 26–32 kb which is currently identified as the largest RNA virus genome size^7^. The SARS-CoV-2 genome comprises 10 functional ORFs; ORF1ab which encodes the viral replication and transcription complex (RTC), 4 structural proteins—spike (S) protein, nucleocapsid (N) protein, envelope (E) protein and membrane (M) protein, and 5 accessory proteins—ORF3a, ORF6, ORF7a, ORF7b and ORF8. ORF1ab comprises two-thirds of the viral genome and encodes two polyproteins (PP1ab and PP1a). PP1ab and PP1a are then cleaved into 16 non-structural proteins nsp1–16; nsp1 (leader protein), nsp2–11 which provide supporting functions for the RTC, RNA-dependent RNA polymerase (nsp12), helicase (nsp13), 3′- to 5′ endonuclease (nsp14), endoRNase (nsp15) and 2′-O-ribose methyltransferase (nsp16). Viral entry into infected cells depends on interaction between the surface spike S protein with angiotensin-converting enzyme 2 (ACE2) host cellular receptor, followed by cleavage of S protein between the S1 and S2 domains, and subsequent internalization of the virus inside the cell. The functions of the S1 and S2 domain is to mediate the binding and downstream membrane fusion, respectively^8^. A subdomain of S1 folds independently and acts as a receptor binding domain (RBD)^9^, binding with high affinity to ACE2. The immune-dominance of the RBD makes it the primary target of natural and vaccine-elicited immunity^7^.

Viral genome sequencing provides a powerful approach to monitor introductions into a country and anticipate spread and evolution of the virus. Certain variants of concern have been defined by the world health organization (WHO) as variants causing increased transmissibility, virulence, or reduced vaccine effectiveness. These variants have notable mutations affecting the spike protein, in addition to mutations in other protein coding sequences. Genetic variations in viral genome can result in a better or worse prognosis, thus studying these mutations is critical to enhance patients’ outcome^10^.

Three software and databases were developed for the real-time tracking of the SARS-CoV-2 evolution through analysis of genomic sequences and the assignment of phylogenetic clades and lineages; Nextstrain, Global Initiative on Sharing Avian Influenza Data (GISAID) and Phylogenetic Assignment of Named Global Outbreak Lineages (PANGO). Analyses done for the S-protein on more than 28,000 spike gene sequences revealed the emergence of a non-synonymous mutation at position 614 (D614G) that was rare before March 2020 and then became more common as the pandemic spread by June 2020^11^. Three other mutations accompanied the D614G substitution: a synonymous/silent C to T mutation at position 3037 in ORF1ab, a C to T mutation at position 241 in the 5` untranslated region and a nonsynonymous C to T mutation at position 14,408 in ORF1ab resulting in mutation P3715L (or P314L) in the hydrophobic cleft near the active site of the RNA-dependent RNA polymerase gene (RdRp/nsp12)^12^.

In this study, 110 SARS-CoV-2 viral isolates from Kasr Al-Aini Hospital and the Children’s Cancer Hospital Egypt (CCHE 57357) were sequenced using short read sequencing of total RNA content. The genomic variation and phylogenetic diversity of SARS-CoV-2 were investigated, as well as mutation patterns and correlation with clinical severity. Finally, co-occuring mutations were investigated and several groups of co-occurring mutations were identified.

## Materials and methods

### Ethical and IRB approval

All procedures performed in the study involving human participants were in accordance with the ethical standards of the institutional research committee of CCHE 57357 and with the 1964 Declaration of Helsinki and its later amendments or comparable ethical standards. A signed informed consent was obtained from the patient (or patient’s guardian for pediatric patients) as an assent to participate in the current study, all of which were approved by the Institutional Review Board at CCHE 57357.

### Sample collection and RNA extraction

Naso-pharyngeal swabs were collected in viral transport media and RNA was extracted using QIAamp® Viral RNA Mini kit (Qiagen). Confirmatory qualitative commercial RT-PCR kits were used for diagnosis and screening (depending on critical availability during the outbreak).

### Library preparation and next generation sequencing

Library preparation was performed using the TruSeq stranded total RNA (Illumina, USA). Samples were then normalized, pooled and subjected to 150-base paired-end reads sequencing using Illumina NextSeq system with a minimum of 2.4 Gb sequencing depth per sample.

### Raw reads processing and mapping

Reads were processed in which low reads quality were filtered out using Trimmomatic^13^ followed by host reads removal through mapping trimmed reads against *Homo sapiens* genome reference (GRCh38) using Burrows-Wheeler Alignment tool (BWA) mem^14^ then unmapped reads were extracted using SAMtools^15^. Unmapped reads were mapped later using BWA mem to Wuhan-Hu-1 (MN908947.3) reference genome. Both mapping runs were aligned using paired-end mode and default parameters. Mapped reads were sorted and indexed using SAMtools.

### Variant detection

Variant calling was performed using Lofreq V2.1.2^16^ starting with realign of aligned reads and indel quality assignment using Viterbi and indelqual commands from LoFreq package. Finally, LoFreq was used to call low-frequency variants.

Generated variants were filtered using LoFreq filter and BCFtools^17^ with default parameters. Filtered variant were annotated with SnpEff^18^ and extracted using SnpSift^19^. Spearman correlation coefficient analysis between selected mutations using cor function in R 4.1.2^20^ and corrplot R package^21^.

### Phylogenetic analysis

Consensus sequence for each sample was computed using mpileup and consensus tools from Bcftools. MAFFT was used to perform multiple sequence alignment^22^ using the following parameters (--6merpair --maxambiguous 0.05 --addfragments) followed by phylogenetic tree calculation using IQ-TREE^23^ which was visualized by iTol^24^.

### Clade and lineage assignment

Finally, clade assignment were performed using Ultrafast Sample placement on Existing tRee (UShER)^25^ and Nextclade^26^. Downstream analysis were performed in R^20^ in which heatmap computed using ComplexHeatmap package^27^, phylogenetic tree visualized using ggtree^28^ and Nextclade online tool (https://clades.nextstrain.org/)^26^.

## Results

### Study population and clinical parameters

A total of 110 diagnosed positive COVID-19 patients were included in the study; 13 from Kasr Al-Aini Hospital and 97 from the outpatient COVID clinic at the CCHE 57357. Samples were collected in the period between May 2020 and January 2021, and included 60 males (54.5%), and 50 females (45.5%). 88 patients were adults (average age 39.6), and 22 were pediatric patients (average age 10.2). Clinical severity ranged from mild (60.9%, n = 67) to moderate (24.6%, n = 27) and severe (14.5%, n = 16), resulting in hospitalization in 36 patients (32.7%) and a survival rate of 97.3%. Clinical data is summarized in Table 1.

**Table 1:** Demographic data of patients from which SARS-CoV-2 samples were isolated.

Clinical variables	Groups	N (%)
Gender	Male	60 (54.5)
	Female	50 (45.5)
Age (adult)	Mean = 39.6 (SD = 15.4)	88 (80)
Age (pediatric)	Mean = 10.2 (SD = 6.3)	22 (20)
Clinical severity	Mild	67 (60.9)
	Moderate	27 (24.6)
	Severe	16 (14.5)
Hospitalisation	Yes	36 (32.7)
	No	74 (67.3)
Status	Alive	107 (97.3)
	Died	3 (2.7)

### Phylogenetic analysis of our samples

**Figure 1:**
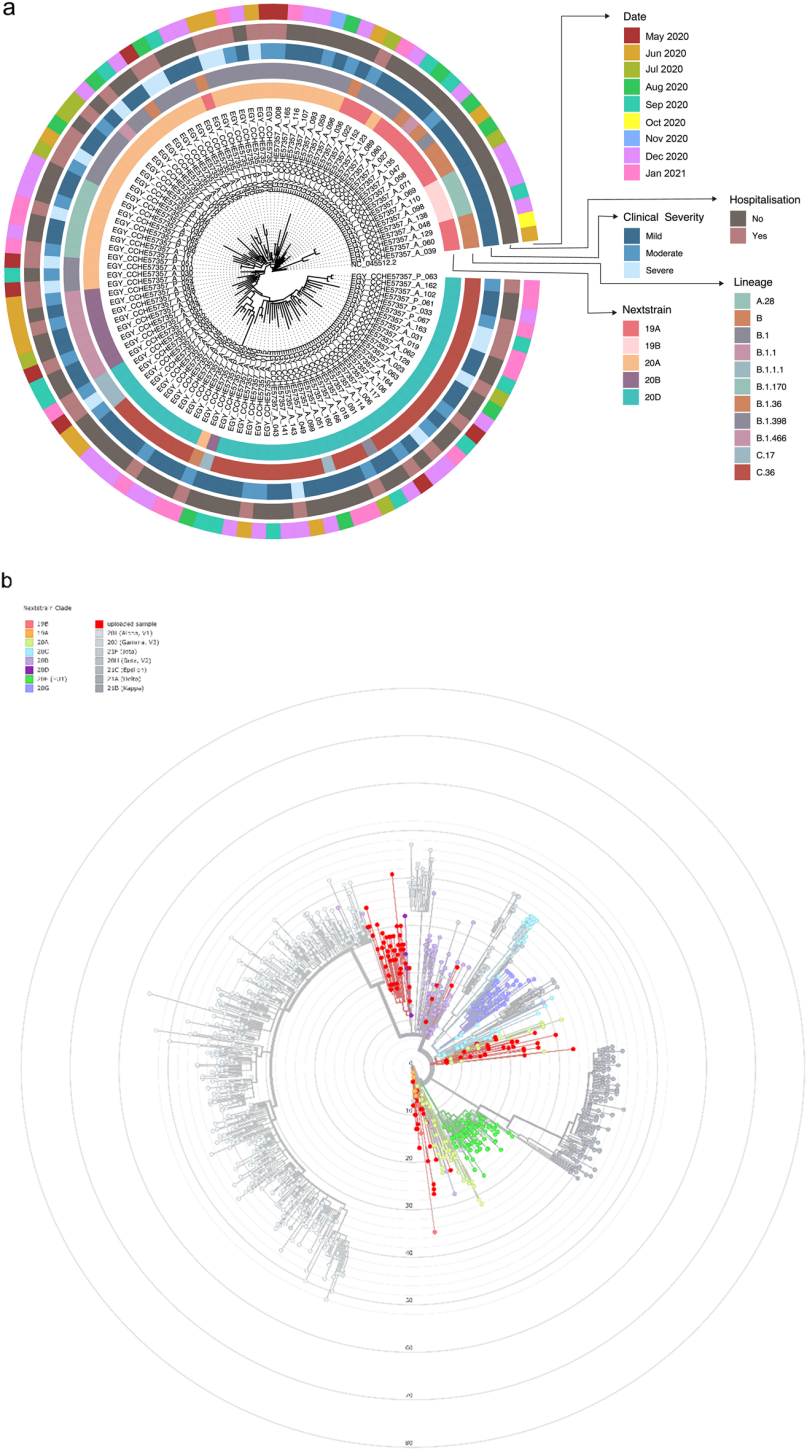
Phylogenetic analysis of 110 SARS-CoV-2 samples included in this study. (**a**) Maximum likelihood phylogenetic tree of 110 SARS-CoV-2 sequences and Wuhan-Hu-1 reference sequence. Visualization of the tree was done using ggtree R package^28^. For each sample, date of sample collection, clinical severity, hospitalization, nextstrain clade and PANGO lineage are indicated by the circular color strip around the tree according to the legend. (**b**) 110 sequenced samples included in this study were placed on Nextclade https://clades.nextstrain.org/^26^. The circles represent the sequences from our study in comparison with published sequences from all over the world. Nextstrain clades are broken down according to the indicated color codes.

To determine phylogenetic characteristics of our SARS-CoV-2 isolates, we performed maximum likelihood tree based on aligned full length sequence of our genomic sequences using IQ-tree and Nextclade online tool (https://clades.nextstrain.org/) (Fig. 1). Nextstrain clade, PANGO lineage, date of sampling, hospitalization and clinical severity are color indicated on the phylogenetic tree in Fig. 1a. Nextstrain classifies strains as follows; 19A is considered the parent strain with no mutations compared to the Wuhan-1 reference strain, *C8782T* and *T28144C* (ORF8 L84S) define clade 19B, and *C3037T*, *C14408T* (ORF1ab P4715L) and *A23403G* (S D614G) define clade 20A. Clades 20B and 20D are derived from clade 20A and have further clade defining mutations; *GG28881-28882AA* (N R203K) and G28883C (G204R) for clade 20B and *C4002T* and *G10097A* (ORF1ab T1246I and G3278S) for clade 20D. Most of our samples belong to Nextstrain clades 20A and 20D, 34.5% and 40% respectively (Fig. 1a, b) and are equally distributed across our sampling dates from May 2020 to January 2021. Clade 19A and 19B appears in our samples mid and late 2020 respectively. Clade 20B disappears by end of 2020 (Supplementary Fig. 1).

### Unique mutation profiles

**Figure 2:**
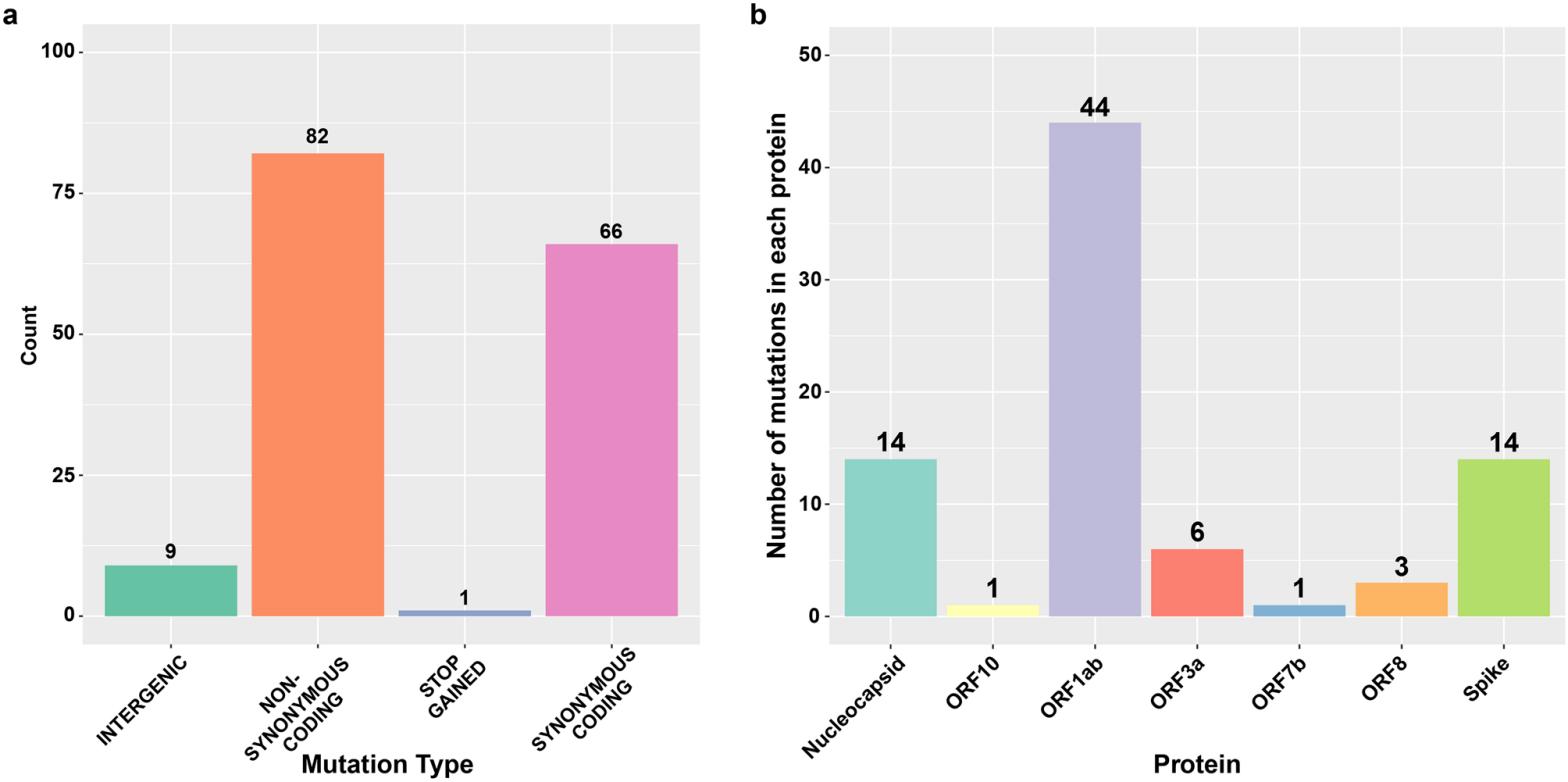
Bar charts showing mutation types and mutations affecting protein. (**a**) Shows different types of mutations detected in the samples. (**b**) Mutations affecting coding regions; nonsynonymous coding and stop codon gain, are divided according to affected protein.

For mutation analysis, samples having mutations with coverage of less than 10X were excluded, this resulted in 71 samples. The SARS-CoV-2 sequences in our study were diverse, and included several mutations. We found 549 mutations in our strains, compared to Wuhan-Hu-1 reference (NC_045512.2) strain, including 294 non-synonymous (amino-acid changing), 224 silent mutations (non-amino acid changing), 24 mutations in intergenic regions, 4 indels and 3 stop codon gained. For further analysis, we excluded mutations that were only reported in one sample. This resulted in 9 mutations in intergenic regions, 82 non-synonymous mutations in coding regions, 66 synonymous (non-amino acid changing mutations), 1 stop codon gained, and 2 indels (Fig. 2a). The non-synonymous coding mutations are described in Fig. 2b, and were distributed across 7 ORFs, 44 in ORF1ab, 14 in the spike encoding gene, 14 in the nucleocapsid encoding gene, 6 in ORF3a, 3 in ORF8, and 1 in each ORF7b and ORF10 (Fig. 2b).

**Figure 3:**
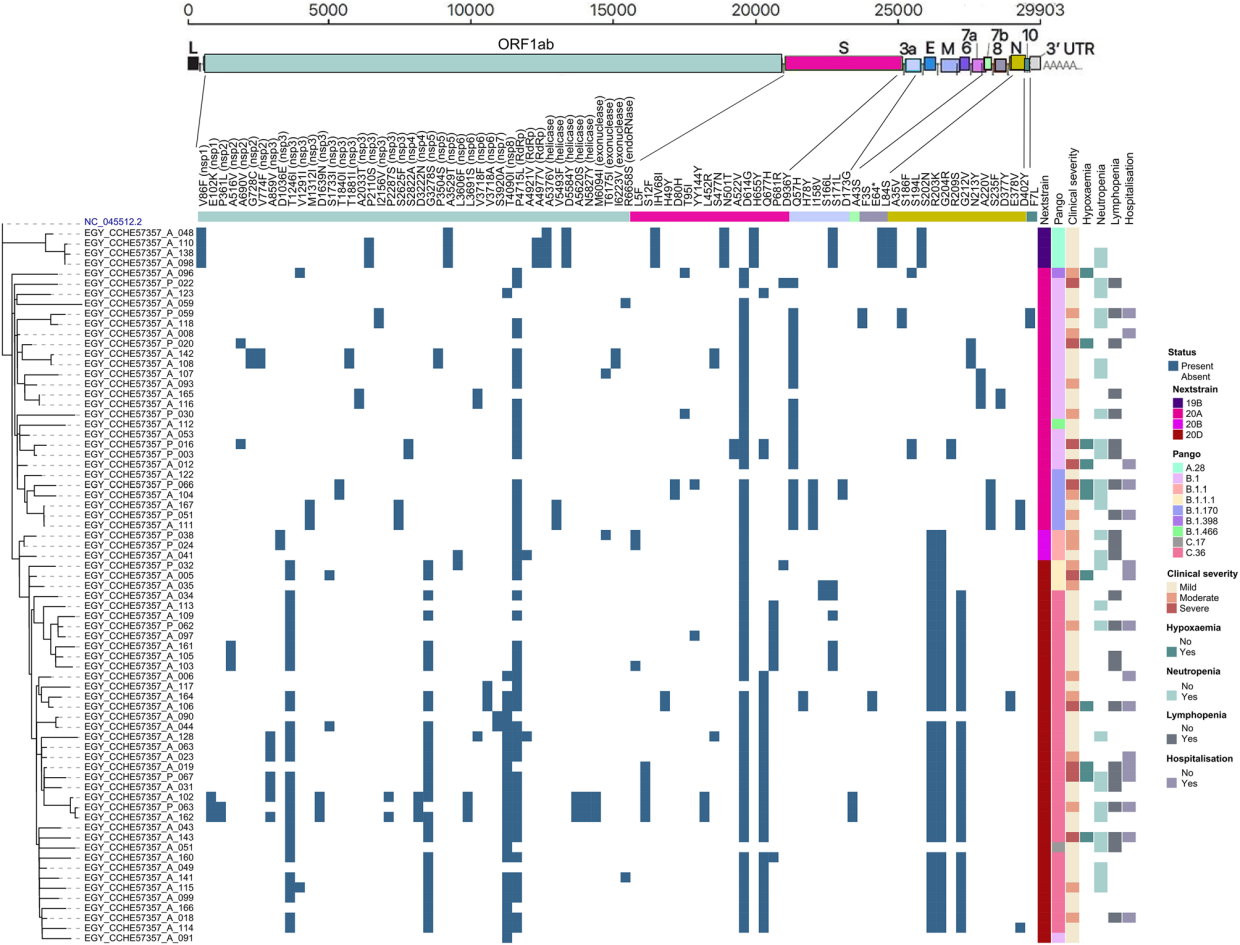
Complex heatmap showing mutations in each sample and clinical data of patients from which samples were isolated.

Certain patterns of mutations appeared in our samples (Fig. 3). Samples belonging to Nextstrain clade 19B also belonged to PANGO lineage A.28 and showed all its characteristic mutations (i) in ORF1ab; V86F (leader protein), A3529T (nsp5), A4977V (RdRp), A5376V and D5584Y (helicase), (ii) in spike; IHV68I, N501T and H655Y, (iii) in ORF3a; S171L, (iv) in ORF8; L84S, and (v) in nucleocapsid; A35V and S202N. These samples also showed mutations not related to their PANGO lineage or Nextstrain clade; ORF1ab P2110S (nsp3) and A4977V (RdRp). Samples belonging to Nextstrain clade 20A and its derivatives 20B and 20D, showed clade characteristic mutations S D614G and ORF1ab P4715L. ORF3a Q57H was prevalent in samples belonging to Nextstrain clade 20A (PANGO lineage B.1, B.1.170 and B.1.466) although not a defining mutation for this clade. Samples belonging to Nextstrain clade 20B and its derivative clade 20D showed its characteristic mutations; nucleocapsid R203K and G204R. Samples belonging to Nextstrain clade 20D showed two additional clade characteristic mutations; ORF1ab T1246I (nsp3) and and G3278S (nsp5) and those of which belonged to PANGO lineage C.36 also showed nucleocapsid G212V. Samples belonging to PANGO lineage B.1.170 showed ORF3a I158V in addition to the clade-defining mutation in nucleocapsid S235F.

### Clinical correlation of mutations

To analyze correlations between mutations and clinical symptoms, we excluded pediatric patients (14 samples with prefix EGY_CCHE57357_P_) as cancer co-morbidity clearly affects clinical presentation (93% moderate or severe clinical symptoms in pediatric cancer patients vs. 26% in adults). Of the 57 adult patients included in the analysis, 42 showed mild clinical symptoms, 10 showed moderate clinical symptoms and 5 showed severe clinical symptoms. Four mutations were correlated with moderate or severe clinical symptoms (Pearson’s correlation coefficient, *p*value < 0.05); *C21707T*, *C25624T*, *G28083T* and *A29406T* which result in amino acid changes in spike H49Y, ORF3a H78Y, ORF8 E64stop and nucleocapsid E378V respectively.

### Patterns of mutation correlation clusters

**Figure 4:**
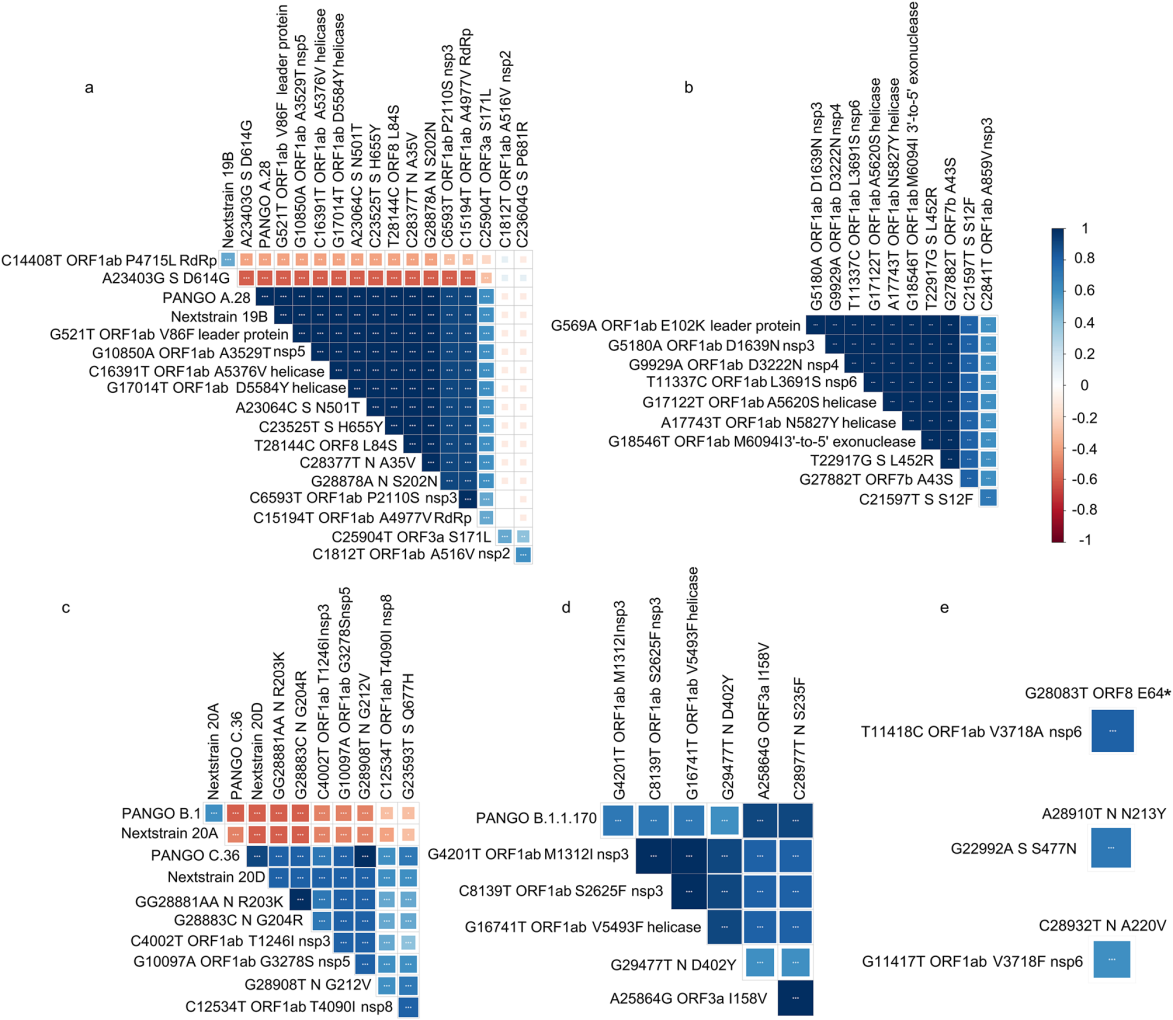
Correlations between different mutations shown by linkage disequilibrium.

Correlation analysis to detect co-occurring non-synonymous mutations was performed. Four major groups of co-occuring mutations were found with Spearman correlation coefficient > 0.6, *p*value < 0.01 (Fig. 4). The first group (Fig. 4a) comprised 14 mutations that were prevalent in PANGO lineage A.28 and Nextclade 19B and are highly correlated. These mutations comprised known clade/lineage defining mutations such as ORF1ab V86F (leader protein), A3529T (nsp5), A4977V (RdRp), A5376V and D5584Y (helicase), in Spike IHV68I, N501T and H655Y, in ORF8 L84S, and in nucleocapsid A35V and S202N, in addition to P2110S (nsp3), ORF3a S171L and Spike P681R. These mutations were negatively correlated to ORF1ab P4715L and Spike D614G. The second group of mutations (Fig. 4b) included 11 mutations with; 8 in ORF1ab E102K (leader protein), A859V and D1639N (nsp3), D3222N (nsp4), L3691S (nsp6), A5620S and N5827Y (helicase), M6094I (exonuclease), two mutations in spike L452R and S12F, and one mutation in ORF7b A43S. The third group of mutations (Fig. 4c) contained seven mutations that were defining mutations for PANGO lineage C.36 and Nextstrain clade 20D, and prevalent to a lesser extent in PANGO lineage B.1 and Nextstrain 20A. These mutations are as follows; three mutations in the nucleocapsid R203K, G204R and G212V, three in ORF1ab; T1246I (nsp3), G3278S (nsp5) and T4090I (nsp8) and one in spike Q677H. The last group of correlated mutations (Fig. 4d) comprised six mutations that were highly prevalent in PANGO B.1.1.170; three in ORF1ab M1312I (nsp3), S2625F (nsp3) and V5493F (helicase), two in nucleocapsid S235F and D402Y, and one in ORF3a I158V. Three single sets of co-occuring mutations are shown in Fig. 4e; ORF1ab V3718A (nsp6) with ORF8 E64stop, spike S477N with nucleocapsid N213Y, and ORF1ab V3718F with nucleocapsid A220V.

## Discussion

Since the emergence of SARS-CoV-2 in China at the end of 2019, the virus has acquired numerous mutations resulting in re-infection and the appearance of new waves in the pandemic. Fortunately, while some mutations have increased virus infectivity, overall it is believed to have evolved into a milder phenotype, in part because of diligent vaccination programs employed by most countries^29,30^.

The available data on the database of GISAID provides about 11.7 million SARS-CoV-2 virus sequences that help in analyzing the genetic diversity in infectious disease epidemiology. For understanding the determinants and patterns of the global spread of SARS-CoV-2 virus, two PANGO lineages were identified as A and B that include sub lineages as A.1, B.1 and A.1.1. Phylogenetic assessment of genomic sequences of our samples revealed they belong to five clades: 19A (B.2), 19B (A.1-A.6), 20A (B.1), 20B and 20D (B.1.1). PANGO lineages B.1.170, B.1.466, C.17 and C.36 were mainly found in Egypt.

In this study, samples collected in Dec 2020 belonged to clade 19B from patients suffering mild symptoms with some reported neutropenia. Samples belonging to clade 20A were collected from May 2020 to January 2021 with all levels of clinical severity ranges from mild to severe symptoms and reports of hypoxemia, neutropenia, and lymphopenia in some patients. Clade 20B circulated from May to August 2020 with patients showing mild to moderate clinical severity and reports of neutropenia and lymphopenia in some. The last clade 20D circulated from May 2020 to January 2021 with patients showing all levels of clinical severity from mild to severe and reports of neutropenia, lymphopenia, and hypoxemia in some patients.

Clade 19B appears late in our samples and harbors D614 in the spike protein. This is in contrast to studies showing the D614 as the original ancestor, and G614 appearing late in the pandemic. D614G occurs at the B-cell epitope, and was reported to enhance the viral replication in human lung epithelial cells and primary human airway tissues thus increasing the infectivity and stability of virions^12^ and was speculated to reduce the efficacy of the vaccines. Our results show an inverse correlation between the occurrence of D614G which occurs in most of the strains, belonging to clades 20A, 20B and 20D and other mutations in the spike N501T, H655Y and IHV68I, which appear in strains belonging to clade 19B.

PANGO lineage A.28 was mostly reported in France, and harbored many key mutations in the spike protein; N501T, H655Y and IHV68I. The N501T spike mutation was predicted to increase the ACE2 binding^31^. IHV68I (or del 69–70) was also reported in the Alpha variant (B.1.1.7) and interferes with viral PCR test accuracy^32^. Other notable spike mutations were reported in our samples. S477N, which lies in the RBD domain, was only found in a few samples in our study and was reported to slightly increase the ACE2 binding. P681H mutation, which is near the furin cleavage site, was found in eight samples in our study belonging to Nextstrain clade 20D and PANGO lineage C.36, and is considered one of the clade defining mutations for the highly contagious delta variant—Nextstrain clade 20I. S477N and P681H were found to confer resistance to antibody therapy^29,33^, whereas contradictory reports were reported for IHV68I^34,35^. A study on 176 viral genome sequences from Egypt showed mutation patterns similar to those found in our data^36^.

In our study, we identified four mutations with association to moderate and severe clinical severity, spike H49Y, ORF3a H78Y, ORF8 E64stop and nucleocapsid E378V. ORF8 gene was reported to be involved in the innate immunity evasion^37^, and similar to our data E64stop mutation was associated with severity of clinical symptoms^38^.

In conclusion, through SARS-CoV-2 viral sequencing, our study identified several lineages circulating in Egypt between May 2020 and January 2021. We identified a large range of mutations throughout the SARS-CoV-2 genome, including four mutations, spike H49Y, ORF3a H78Y, ORF8 E64stop and nucleocapsid E378V, that were associated with higher disease severity. Additionally, we identified several mutation groups that were associated together and in specific clades. These results could provide a starting point for in vitro and in vivo analysis for the functions of these mutations, and are vital for virus tracking and the development of novel vaccines.

## Supplementary Information

Supplementary Information.

## Data Availability

All data generated and analyzed during this study are included in this article and published online on NCBI with BioProject ID PRJNA818451 https://www.ncbi.nlm.nih.gov/bioproject/PRJNA818451.
