# Supplementary material for: SARS-CoV-2 genome variations and evolution patterns in Egypt: a multi-center study
Source: Sci Rep. 2022 Aug 25;12:14511. doi: 10.1038/s41598-022-18644-4 (PMC9403952; doi:10.1038/s41598-022-18644-4)
Supplement: Supplementary file 1 — Supplementary Information. [file 41598_2022_18644_MOESM1_ESM.docx]

## Supplementary Figure 1: Clade distribution across sample dates

20D

20B

20A

19A

19B
